# Immunomodulatory factors CRP/albumin ratio and NLR predict post-spinal surgery infection

**DOI:** 10.3389/fsurg.2025.1627141

**Published:** 2025-09-11

**Authors:** Chang Yuping, Wei Rong, Li Fengjuan, Liu Chunhua, Dong Zhenghui

**Affiliations:** Departments Outside the Spine, Sixth Affiliated Hospital of Xinjiang Medical University, Urumqi, Xinjiang Uygur Autonomous Region, China

**Keywords:** postoperative spinal infection, neutrophil/lymphocyte ratio, C-Reactive, predictive value, spinal infection

## Abstract

**Objective:**

The objective of this work is to investigate the predictive value of the neutrophil-to-lymphocyte ratio (NLR) combined with the C-reactive protein/albumin (CRP/ALB) ratio for postoperative infections in patients undergoing spinal surgery.

**Methods:**

According to the inclusion criteria, 380 patients who underwent spinal surgery treatment in the Sixth Affiliated Hospital of Xinjiang Medical University between January 2020 and December 2023 were retrospectively screened and divided into two groups based on whether they were infected after surgery. The two groups were 1. infected group (*n* = 79) and 2. uninfected group (*n* = 301). The following variables were reviewed in both groups: gender, age, body mass index, hypertension, diabetes mellitus, coronary artery disease, history of smoking, history of alcohol consumption, duration of surgery, site of surgery, presence of blood transfusion, presence of internal fixation, NLR and CRP/ALB ratio. A one-way analysis was performed on these factors, and those with a *P* < 0.05 were replaced with a binary logistic analysis in order to investigate the factors associated with postoperative infection.

**Results:**

Comparative analysis revealed significant between-group differences (*P* < 0.05) in age, diabetes status, operative duration, blood transfusion, internal fixation use, postoperative NLR, and CRP/ALB ratio. Binary logistic regression identified six independent risk factors: advancing age (OR = 1.145, 95% CI 1.098–1.203), prolonged operative time (OR = 1.020, 95% CI 1.010–1.030), intraoperative blood transfusion (OR = 2.941, 95% CI 1.245–7.211), internal fixation placement (OR = 8.022, 95% CI 2.710–25.615), elevated postoperative NLR (OR = 1.870, 95% CI 1.531–2.324), and increased CRP/ALB ratio (OR = 2.178, 95% CI 1.673–2.943). All associations reached statistical significance (*P* < 0.001 unless specified).

**Conclusion:**

The results indicate that age, duration of surgery, blood transfusion, internal fixation, postoperative NLR and postoperative CRP/ALB are risk factors for the development of infection after spinal surgery. Surgeons should perform a thorough assessment of their patients in order to more accurately predict their likelihood of infection and to provide a basis for individualised treatment plans to reduce the risk of postoperative infections.

## 1 Introduction

In recent years, with the advancement of medical technology, most patients with spinal disorders can be treated by surgery, which significantly improves their quality of life. However, due to the large surgical trauma, long operation time, and the need to implant exogenous fixation, the risk of postoperative infections in patients is relatively high (1). Some studies have shown that the high incidence of postoperative infections in spinal surgery patients not only prolongs the hospitalisation time of the patients, which is not conducive to wound healing, but may also lead to secondary surgery, which increases the difficulty of treatment and prognosis of rehabilitation, and has a serious impact on the patients' physical health and quality of life (2, 3). Neutrophils to lymphocytes ratio (NLR) can reflect the balance between inflammation-activated neutrophils and regulatory lymphocytes, and is one of the common indicators for diagnosing infectious diseases (4). Studies have shown that NLR demonstrates significant clinical value in the diagnosis of early infection in fracture patients undergoing surgery (5). In addition, CRP/ALB ratio, two proteins synthesised by liver cells, have different physiological functions. Specifically, CRP levels increase significantly when the body is exposed to microbial attack or tissue damage, while ALB, the most abundant protein in the serum, is mainly used to assess the nutritional status of patients. However, recent studies have also found an association between ALB and the inflammatory response triggered by infection. Therefore, the ratio of CRP/ALB ratio can be regarded as an indicator of inflammatory status in the body (6). Currently, the application of NLR and CRP/ALB ratio in predicting early infection after open fracture and bone arthroplasty has been investigated, however, they have been relatively less studied in the prediction of postoperative infections in patients with spinal disorders (7–9). In this study, we retrospectively analysed the clinical data of patients with osteoporotic vertebral compression fractures from January 2020 to December 2023, and used logistic regression to analyse the predictive risk factors for postoperative infection after spinal surgery, in order to provide effective preventive and therapeutic measures to prevent postoperative infections and improve surgical outcomes.

## 2 Subjects and methods

### 2.1 Design

Retrospective comparative test.

### 2.2 Time and place

The trial was completed from January 2020 to December 2023 in the Department of Orthopaedics, the Sixth Affiliated Hospital of Xinjiang Medical University.

### 2.3 Inclusion criteria

i. Age ≥18 years old; ii. Diagnosed with Degenerative spinal disease by clinical and imaging examination; iii. Including the following procedures: Spinal Stenosis Decompression; Discectomy for Herniated Disc; Posterior Lumbar Interbody Fusion with Internal Fixation for Spondylolisthesis, among others; iv. Data and follow-up results were complete. v. Passed by the Ethics Committee of the Sixth Affiliated Hospital of Xinjiang Medical University, NO.20240710-01. All patients signed an informed consent form prior to surgery, which included the following clause: “Anonymized medical data may be used for medical research purposes”.

### 2.4 Exclusion criteria

i. patients with a history of previous spinal surgery; ii. patients with pathological fractures caused by tumours or infections; iii. patients with severe neurological, psychiatric, or basic illnesses or other inability to cooperate with pain assessment; iv. patients with a body mass index >35 kg/m^2^; and v. patients with coagulation disorders.

### 2.5 General information

380 patients with spinal disorders who underwent surgery in our hospital from January 2020–December 2023 were selected for the study. They were divided into 301 cases in the uninfected group and 79 cases in the infected group according to whether or not infection occurred within 7 days after surgery. At least two of the following criteria were met for infection: 1. the bacterial culture results of the tissues were positive; 2. the tissues showed a significant increase in neutrophils under high magnification; and 3. the presence of signs and symptoms of infection, such as redness, fever, tenderness, swelling, pain, etc., and the infection was also supported by laboratory tests and imaging tests. All spinal surgeries were performed at the Spinal Surgery Center (Provincial Referral Center for Spinal Disorders) of the Sixth Affiliated Hospital of Xinjiang Medical University. The procedures were conducted by a team of four attending physicians and senior specialists with spinal surgery qualifications (each possessing >10 years of spinal surgery experience), strictly adhering to standardized surgical protocols. The scope of treatment includes: Spinal Stenosis Decompression; Discectomy for Herniated Disc; Posterior Lumbar Interbody Fusion with Internal Fixation for Spondylolisthesis, among others.

### 2.6 Test method

The human CRP and ALB enzyme-linked immunosorbent assay (ELISA) kits (Item No.: H-EL-CRP, HEL-ALB) were purchased from Shanghai Zeye Biotechnology Co. The ELX800 automatic enzyme labeller was purchased from BIO-TEK, USA. Peripheral venous blood was drawn on an empty stomach 24 h before and 24 h after the operation, and was divided into two portions; one portion was tested for blood routine using the automatic blood cell analyser and its accompanying kit to calculate NLR; the other portion was centrifuged at 2,500 r/min for 10 min to separate serum, and then CRP and ALB levels were measured by using the CRP and ALB ELISA kits and the enzyme marker to calculate CRP/ALB ratios.

### 2.7 Evaluating indicator

General information was collected, including gender, age, body mass index (BMI), past history, including hypertension, diabetes mellitus, coronary artery disease, smoking history, and history of alcohol consumption; surgical information, including time of surgery, surgical site, the presence of blood transfusion, and the presence of internal fixation; and test results, including NLR and CRP/ALB ratio, were recorded.

Infection definition: According to the Guidelines of the Centers for Disease Control and Prevention (CDC) and Infectious Diseases Society (IDSA).

#### 2.7.1 Superficial surgical site infection (SSI)

Infection within 30 days post-surgery involving only skin/subcutaneous tissue, with ≥1 of:

• Purulent drainage from the incision.
• Positive aseptic culture.
• Symptoms (pain/swelling/erythema) + deliberate opening by surgeon.

#### 2.7.2 Deep/implant-related infection

Infection within 90 days involving deep soft tissues (fascia/muscle) or implants, with ≥1 of:

• Purulent drainage from deep tissue.
• Abscess/infection seen during reoperation/histopathology.
• Symptoms (fever/localized pain) + positive deep tissue/implant culture.

### 2.8 Statistical methods

SPSS26.0 statistical software was used to statistically analyse the patients' gender, age, BMI, past history, surgical data, and test results. Firstly, one-way factor analysis was performed, and the count data were expressed as cases (rate) by *x*^2^ test, and the measure data were expressed as mean ± standard deviation by *t* test. Among the results of one-way analysis, the factors with *P* < 0.05 were selected for multifactorial regression logistic analysis, in which the clinical characteristics of the patients were used as the independent variables and postoperative infection as the dependent variable in order to assess the potential risk factors for postoperative infection after spinal surgery, and the difference was expressed as statistically significant with *P* < 0.05.

## 3 Results

### 3.1 Analysis of participant number

380 spinal surgery patients were included, divided into 2 groups according to whether they were infected or not, 79 in the infected group and 301 in the uninfected group, and all of them were entered into the analysis of the results, with no dropout data.

### 3.2 Test flow chart

**Figure 1:**
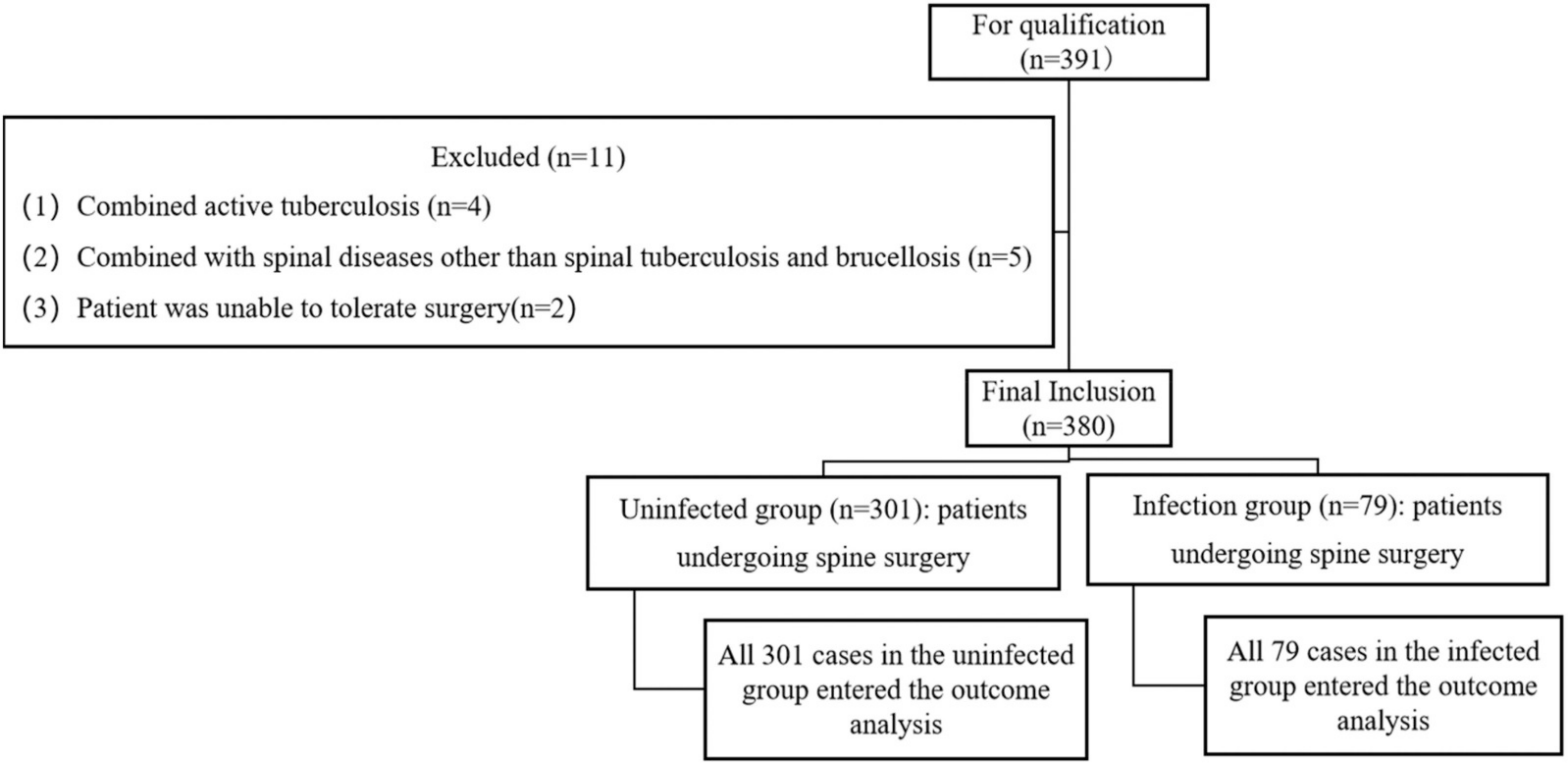
Flow chart of patient assignment.

The group flow chart of the two groups is shown in Figure 1.

### 3.3 A unifactorial analysis of infections occurring after spinal surgery

**Figure 2:**
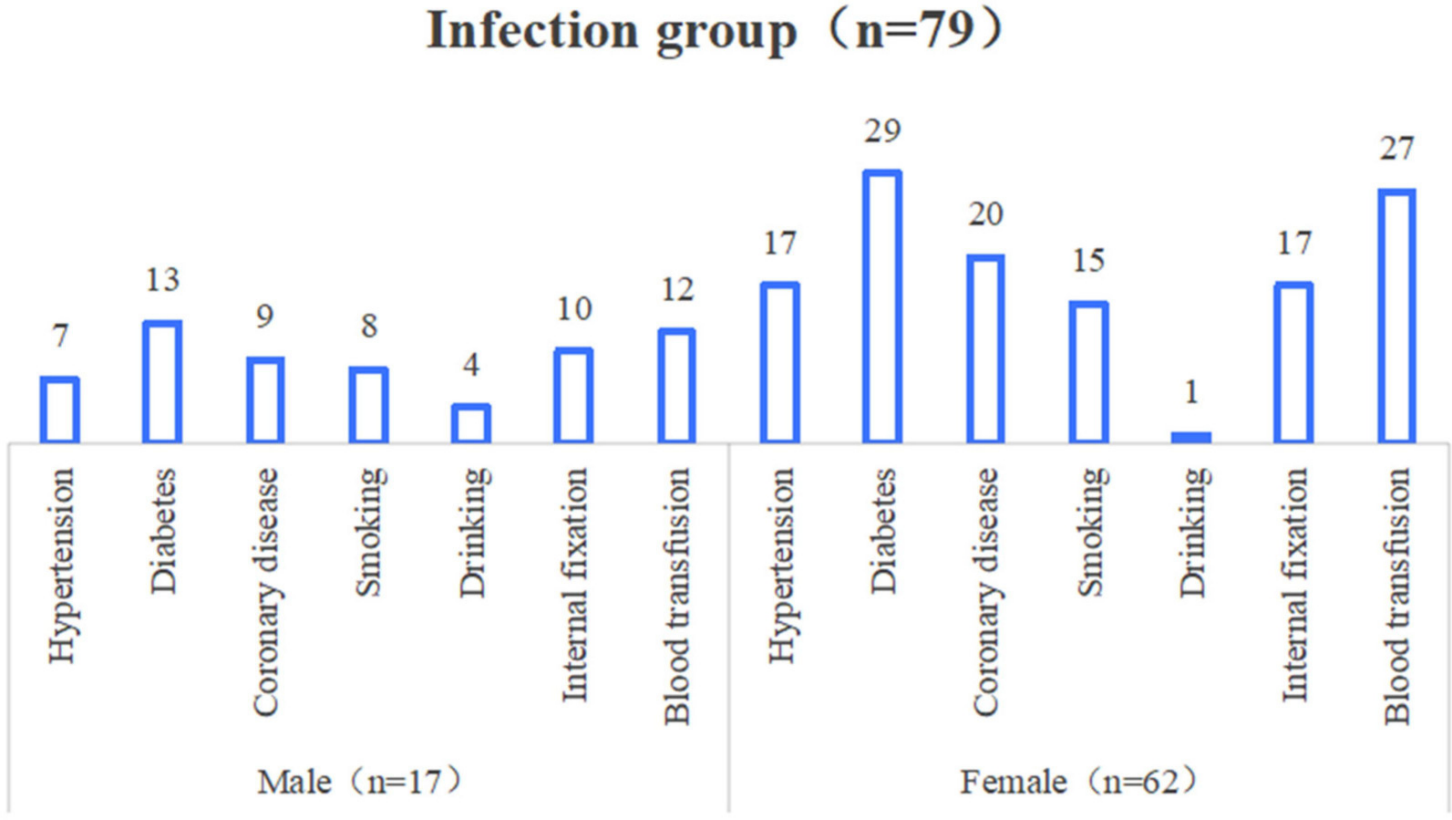
Sex distribution of main indicators in the infected group.

The results of univariate analysis showed that the differences in age, diabetes mellitus, operation time, blood transfusion, internal fixation, postoperative NLR and postoperative CRP/ALB ratio were statistically significant when comparing the two groups (*P* < 0.05); and the differences in comparison of gender, BMI, hypertension, coronary artery disease, history of smoking, history of alcohol consumption, and surgical site were not significant (*P* > 0.05), as shown in Table 1 and Figure 2.

**Table 1:** Results of the univariate analysis of the infection occurring after spinal surgery.

Variables	Total (*n* = 380)	Uninfected group (*n* = 301)	Infection group (*n* = 79)	Statistic	*P*
Age, ($\bar{X}\pm S$)	63.071 ± 13.741	60.405 ± 13.629	73.228 ± 8.382	*t* = −10.401	**<0** **.** **001**
BMI, ($\bar{X}\pm S$)	210.594 ± 109.696	212.087 ± 112.216	204.904 ± 99.306	*t* = 0.554	0.581
Sex, *n*(%)				*χ*² = 0.323	0.570
Male	91 (23.947)	74 (24.585)	17 (21.519)		
Female	289 (76.053)	227 (75.415)	62 (78.481)		
Hypertension, *n*(%)				*χ*² = 1.383	0.240
No	284 (74.737)	229 (76.080)	55 (69.620)		
Yes	96 (25.263)	72 (23.920)	24 (30.380)		
Diabetes, *n*(%)				*χ*² = 7.509	**0** **.** **006**
No	229 (60.263)	192 (63.787)	37 (46.835)		
Yes	151 (39.737)	109 (36.213)	42 (53.165)		
Coronary disease, *n*(%)				*χ*² = 1.490	0.222
No	262 (68.947)	212 (70.432)	50 (63.291)		
Yes	118 (31.053)	89 (29.568)	29 (36.709)		
Smoking, *n*(%)				*χ*² = 2.393	0.122
No	294 (77.368)	238 (79.070)	56 (70.886)		
Yes	86 (22.632)	63 (20.930)	23 (29.114)		
Drinking, *n*(%)					
No	363 (95.526)	289 (96.013)	74 (93.671)	*χ*² = 0.803	0.370
Yes	17 (4.474)	12 (3.987)	5 (6.329)		
Opera-time, ($\bar{X}\pm S$)	151.118 ± 39.293	144.834 ± 38.266	175.063 ± 33.522	*t* = −6.389	**<0** **.** **001**
Operative site, *n*(%)				*χ*² = 0.824	0.662
Cervical vertebra	117 (30.789)	95 (31.561)	22 (27.848)		
Thoracic vertebra	124 (32.632)	95 (31.561)	29 (36.709)		
Lumbar vertebra	139 (36.579)	111 (36.877)	28 (35.443)		
Internal fixation, *n*(%)				*χ*² = 32.615	**<0** **.** **001**
No	326 (85.789)	274 (91.030)	52 (65.823)		
Yes	54 (14.211)	27 (8.970)	27 (34.177)		
Blood transfusion, *n*(%)				*χ*² = 6.425	**0** **.** **011**
No	239 (62.895)	199 (66.113)	40 (50.633)		
Yes	141 (37.105)	102 (33.887)	39 (49.367)		
NLR ($\bar{X}\pm S$)					
Preoperative	2.101 ± 0.781	2.360 ± 0.689	2.292 ± 0.687	*t* = 1.244	0.222
Postoperative	4.804 ± 1.758	4.381 ± 1.554	6.413 ± 1.552	*t* = −10.322	**<0** **.** **001**
CRP/ALB ratio ($\bar{X}\pm S$)					
Preoperative	1.203 ± 0.569	1.139 ± 0.465	1.126 ± 1.247	*t* = 0.039	0.843
Postoperative	2.985 ± 1.371	2.678 ± 1.179	4.154 ± 1.422	*t* = −8.443	**<0** **.** **001**

Bold indicates statistical difference, *P* < 0.05.

*t*, *t*-test; χ², Chi-square test; $\bar{X}\pm S$, Mean ± standard deviation; NLR, neutrophils to lymphocytes ratio; CRP/ALB ratio, C-reactive protein/albumin ratio; BMI, body mass index.

### 3.4 Multifactorial logistic regression analysis of infections occurring after spinal surgery

**Figure 3:**
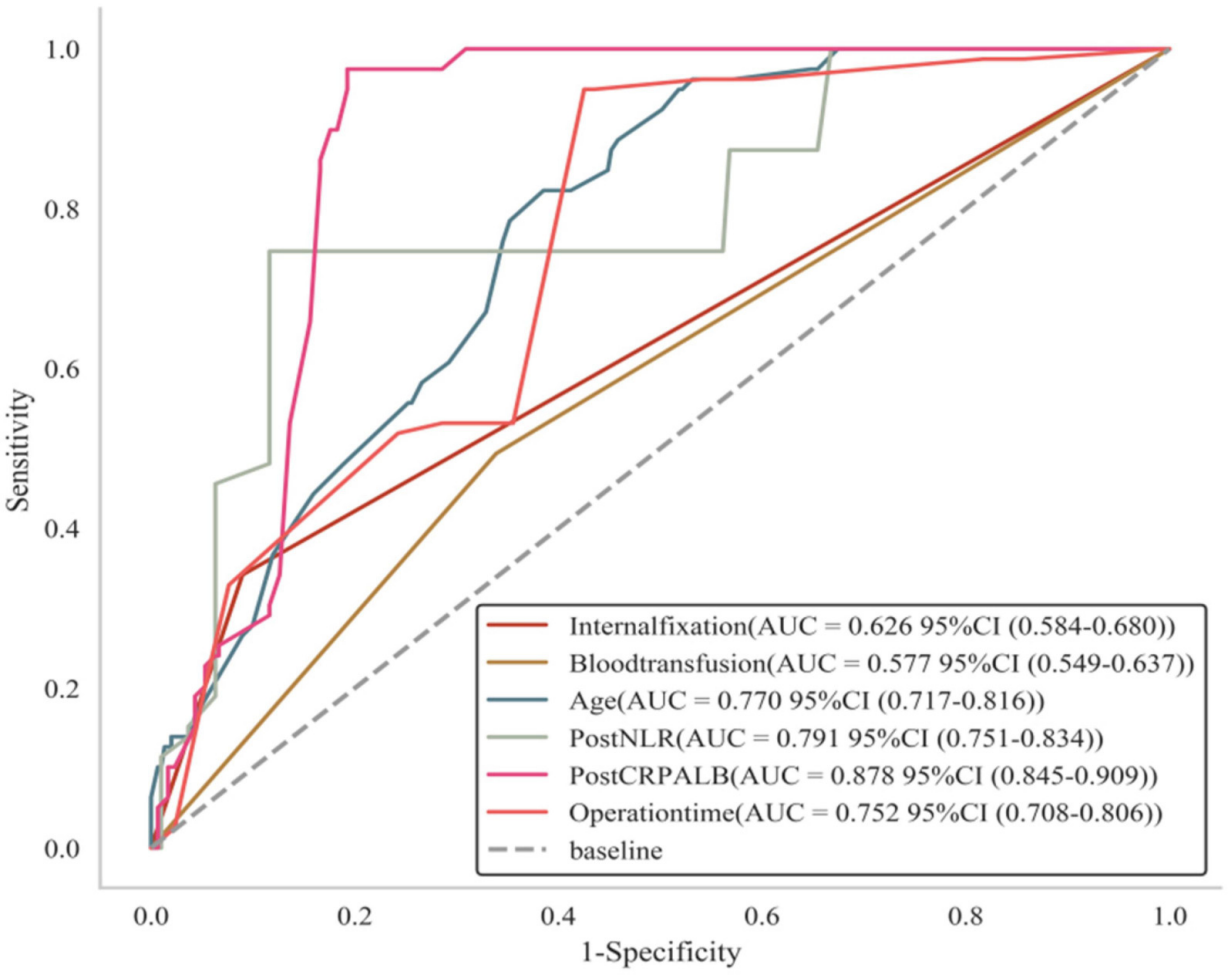
ROC curves of infection occurring after spinal surgery. Age, diabetes mellitus, duration of surgery, blood transfusion, internal fixation, postoperative NLR and postoperative CRP/ALB ratio curves are above the reference line, indicating that all six of these indicators are risk factors for infection after spinal surgery.

A multifactorial logistic regression analysis was performed with the occurrence of infection after spinal surgery as the dependent variable, and seven variables (age, diabetes mellitus, duration of surgery, blood transfusion, internal fixation, postoperative NLR, and postoperative CRP/ALB ratio) screened by univariate analysis as the independent variables. The results showed that age [OR = 1.145, 95% CI (1.098–1.203), *P* < 0.001], operation time [OR = 1.020, 95% CI (1.010–1.030), *P* < 0.001], blood transfusion [OR = 2.941, 95% CI (1.245–7.211), *P* = 0.015], and internal fixation [OR = 8.022, 95% CI (2.710–25.615), *P* < 0.001], postoperative NLR [OR = 1.870, 95% CI (1.531–2.324), <0.001], and postoperative CRP/ALB ratio [OR = 2.178, 95% CI (1.673–2.943), *P* < 0.001] were spinal independent risk factor for developing infection after surgery. See Table 2 and Figure 3.

**Table 2:** Results of the multivariate logistic regression of infection occurring after spinal surgery.

Variables	*β*	S.E	Z	*P*	OR (95% CI)
Intercept	−20.821	2.51	−8.295	**<0**.**001**	0.000 (0.000–0.001)
Age	0.136	0.023	5.842	**<0**.**001**	1.145 (1.098–1.203)
Diabetes	0.727	0.433	1.678	0.093	2.069 (0.893–4.931)
Operation time	0.019	0.005	3.86	**<0**.**001**	1.020 (1.010–1.030)
Blood transfusion	1.079	0.445	2.424	**0**.**015**	2.941 (1.245–7.211)
Internal fixation	2.082	0.569	3.657	**<0**.**001**	8.022 (2.710–25.615)
Postoperativ-NLR	0.626	0.105	5.949	**<0**.**001**	1.870 (1.531–2.324)
Postoperativ-CRP/ALB ratio	0.778	0.142	5.464	**<0**.**001**	2.178 (1.673–2.943)

Bold indicates statistical difference, *P* < 0.05.

OR, odds ratio; CI, confidence interval; NLR, neutrophils to lymphocytes ratio; CRP/ALB ratio, C-reactive protein/albumin ratio.

### 3.5 Predicted value analysis

The results showed that the features of internal fixation, blood transfusion, age, postoperative NLR, postoperative CRP/ALB ratio and operation time, the results showed that the AUC value of the internal fixation feature was 0.626, which had medium predictive ability, with low sensitivity and high specificity; the AUC value of the blood transfusion feature was only 0.577, which had relatively low predictive ability; the AUC value of the age feature was 0.770, which showed good predictive The AUC value for the age feature was 0.770, showing good predictive ability with good sensitivity and specificity; the AUC value for the postoperative NLR feature was 0.791, also with good predictive ability; especially prominent was the postoperative CRP/ALB ratio feature, which had an AUC value of 0.878, with high sensitivity and specificity, indicating its excellent performance in predicting the target; and the AUC value for the surgical time feature was 0.752, with predictive ability is fair. The optimal thresholds for each feature also varied, providing an important reference for clinical decision-making. See Table 3.

**Table 3:** Sensitivity analysis of infection occurring after spinal surgery.

Variables	AUC	Sensitivit	Specificity	Youden index	Optimal threshold
Internal fixation	0.626	0.342	0.910	0.252	1.000
Blood transfusion	0.577	0.494	0.661	0.155	1.000
Age	0.770	0.823	0.615	0.437	67.000
Post-NLR	0.791	0.747	0.884	0.631	6.200
Post-CRP/ALB ratio	0.878	0.975	0.807	0.782	2.670
Operation time	0.752	0.949	0.575	0.524	150.000

## 4 Discussion

In recent years, with the aging of society, the incidence of spinal diseases has continued to climb, prompting more and more patients to prefer surgery as a means of treatment (10). However, factors such as prolonged operation time, the placement of internal fixation, and the decline in patients' physical fitness have all become high-risk factors for postoperative infection (11). Postoperative spinal infections not only significantly elevate the risk of patient mortality rates but also substantially increase healthcare expenditures for both patients' families and society as a whole (12). Therefore, the prevention of postoperative spinal infections has increasingly received widespread attention from all sectors of society. Laboratory indicators play a pivotal role in the diagnosis of postoperative orthopedic infections. Although bacterial cultures of incisional secretions and deep puncture fluids can provide important diagnostic clues, their longer detection period, lower positive rate, and possible contamination during specimen collection may affect the accuracy of diagnostic results (13). Meanwhile, peripheral blood leukocytes and CRP, as inflammatory markers, though they have reference value, face limitations in application due to susceptibility to factors such as immune status and inflammatory responses. In recent years, serum CD64, VEGF, SDF-1, NLR, and the CRP/ALB ratio have been increasingly recognized for their potential in early infection diagnosis. The aim of this study was to explore the application value of these indicators in predicting postoperative infections in patients with spinal disorders, thereby providing a scientific basis for clinicians to implement timely and effective interventions (14).

Neutrophils, lymphocytes, and thrombocytosis are important manifestations of systemic inflammation, and NLR and PLR calculated from the above indicators can more accurately reflect the severity of systemic inflammation, therefore, NLR and PLR have been gradually used in the diagnosis of postoperative spinal infections and their complications (15). NLR is the ratio of neutrophils to lymphocytes, which is a readily available indicator. It can exclude the effects of excessive body fluids, dehydration, and specimen handling (16). NLR is also a reliable indicator for the assessment of conditions such as infections, chronic obstructive pulmonary disease, and cancer (17). In our case, NLR levels were significantly higher in the infected group compared to the non-infected group. The multifactorial lo gistic regression model corrected for confounders still suggested an association between NLR level showed an independent association with postoperative infection. Therefore NLR can be used as a predictor of postoperative spinal infection. The mechanisms may be 1. neutrophils, as an important participant in the immune response to infection, arrive at the site of inflammation under the action of chemokines to phagocytose and kill pathogenic bacteria (18); and 2. lymphocyte apoptosis is increased in the state of immuno inflammation. The NLR can reflect changes in both neutrophil and lymphocyte levels, and thus its changes are more effective in reflecting the presence of infection than a single indicator (19).

CRP is an acute temporal response protein synthesised by the liver, which is an important component of the immune system and enhances phagocytosis by some CRP-expressing receptor macrophages (20). Studies have shown (21) that CRP is significantly associated with postoperative infections and complications. Wei Kewei et al. (22) found in a retrospective study of primary total knee replacement patients that a CRP level of 35 mg/L was the best diagnostic for periprosthetic infection, which could be used as a diagnostic basis for postoperative infection. In a study of postoperative complications in patients with Crohn's disease, Xu Xin et al. (23) showed that serum CRP levels have a predictive value for the occurrence of postoperative infectious complications in the abdominal cavity. In this study, serum CRP was significantly higher in the postoperative complication group than in the uncomplicated group of orthopaedic patients, suggesting that serum CRP levels may be intrinsically related to the occurrence of postoperative complications. CRP/ALB ratio levels are a good reflection of microvascular permeability, and there is a certain correlation between the levels and the degree of capillary leakage, which is one of the pathological features of organic infection (24). However, when a patient develops an infection, the liver synthesises stress response proteins such as CRP, which in turn reduces the amount of ALB synthesis, leading to an increase in CRP/ALB ratio levels (25). Therefore, elevated CRP/ALB ratio level was significantly associated with postoperative spinal infection.

The results of this study show that, based on the sensitivity analysis, Post-CRP/ALB ratio has the strongest predictive power (AUC = 0.878,95% CI: 0.845–0.909), indicating that the combined indicator of inflammation and nutritional status is significantly valuable for predicting outcomes; Age and Post-NLR have AUCs of 0.770 and 0.791, respectively, suggesting moderate predictive power for age and postoperative inflammatory response; Blood transfusion has the lowest AUC (0.577, 95% CI: 0.549–0.637), with its confidence interval approaching the random guess level (0.5), indicating limited predictive value of a single transfusion indicator. Overall, the results suggest that postoperative inflammatory-related indicators (such as Post-CRP/ALB ratio, Post-NLR) and age are core predictive factors, and their application potential needs to be further validated in clinical context. The results of multifactorial logistic regression analysis showed that older age, longer duration of surgery, intraoperative blood transfusion, use of internal fixation, and elevated postoperative NLR and CRP/ALB ratio suggested an increased risk of postoperative infection and the need for timely measures to prevent infection.

This study innovatively demonstrates the synergistic value of the neutrophil-to-lymphocyte ratio (NLR) and C-reactive protein/albumin ratio (CRP/ALB) in predicting postoperative spinal infections, addressing a critical evidence gap in biomarker applications for this field. Distinct from prior orthopedic research, this work is the first to systematically validate the superior predictive efficacy of postoperative CRP/ALB (AUC = 0.878, sensitivity 97.5%, specificity 80.7%) and NLR (AUC = 0.791) in a large-scale spinal surgery cohort (*n* = 380), establishing spine-specific optimal cutoff thresholds of CRP/ALB > 2.67 and NLR > 6.2. Crucially, multivariate regression reveals a unique high-risk architecture inherent to spinal surgery: internal fixation implantation emerged as the strongest independent risk factor (OR = 8.022, *P* < 0.001), which—together with operative duration and intraoperative blood transfusion—constitutes core procedural variables for spinal infection. Meanwhile, postoperative markers of inflammation-nutrition imbalance (CRP/ALB) and immune stress (NLR) are intrinsically linked to the pathophysiological processes triggered by deep-tissue trauma and implant placement. This model not only provides a highly sensitive tool for early infection detection but also establishes a dedicated risk assessment framework integrating surgical interventions and host response, thereby facilitating the development of individualized clinical prevention strategies.

However, this study also had the following limitations: i. it was a retrospective case study, which may have a selective bias of its own, in addition to the fact that it was not possible to radiologically screen all patients undergoing spinal surgery, which may have affected the results; ii. the sample for this study came from only one hospital database, which may have affected the generalisability of the results, and subsequent multi-centre, large-sample studies are still needed; iii. it was only internally validated, with the Lack of external datasets for further validation of the predictive ability of column line plots; iv. Future studies can explore the integration of Post-NLR and Post-CRP/ALB ratio indicators into a composite prediction model to further improve the discrimination ability; and v. In future clinical trials and cohort studies, gender must be analyzed as a key stratification or covariate to avoid masking true efficacy or risk or drawing biased conclusions due to the omission of gender factors. This will help obtain more reliable and generalizable results.

## 4 Conclusion

The results of this study suggest that the risk factors for postoperative spinal infection are multifactorial, including (age, surgery time, blood transfusion, internal fixation, postoperative NLR and postoperative CRP/ALB ratio, which may serve as potential predictors of postoperative spinal infection.

## Data Availability

The raw data supporting the conclusions of this article will be made available by the authors, without undue reservation.
